# Patient-led use of patient-reported outcome measure in self-Management of a Rotator Cuff Injury

**DOI:** 10.1186/s41687-020-00283-w

**Published:** 2021-01-13

**Authors:** Maria J. Santana, Darrell J. Tomkins

**Affiliations:** 1 Departments of Pediatrics and Community Health Sciences, Cumming School of Medicine, University of Calgary, 3280 Hospital Drive NW, TRW Building, 3rd Floor, Calgary, Alberta T2N 4Z6 Canada; 2 Department of Medical Genetics, University of Alberta, Edmonton, Canada

## Abstract

**Introduction:**

The patient is the person who experiences both the processes and the outcomes of care. Information held by the patient is vital for clinical and self-management, improving health outcomes, delivery of care, organization of health systems, and formulation of health policies. Patient-reported outcome measures (PROMs) play an important role in supporting patient’s self-management. This narrative describes a patient-led use of a PROM to self-manage after a rotator cuff injury.

**Methods:**

This is a narrative of a patient who tore the supraspinatus tendon in her right shoulder in an accident. The *Disabilities of the Arm, Shoulder and Hand,* the DASH questionnaire, was used to monitor and self-manage recovery after the accident.

The DASH questionnaire is a self-reported questionnaire that measures the difficulty in performing upper extremity activities and pain in the arm, shoulder or hand. It has been widely used in research studies, but here the patient initiated its use for self-management while waiting for and after rotator cuff surgery. The patient created separate sub-scale scores for function and for pain to answer questions from healthcare providers about her recovery.

**Results:**

There was noticeable improvement over 3 months of conservative treatment, from a high level of disability of 56 to 39 (score changed 17); however, the scores were nowhere near the general population normative score of 10.1. Surgery improved the score from 39 pre-surgery to 28. Post-surgical interventions included physiotherapy, pain management and platelet-riched plasma treatment (PRP). The score was 14 4 weeks post-PRP.

**Conclusions:**

The patient found the DASH useful in monitoring recovery from a rotator cuff injury (before and after surgery). The DASH contributed to communication with healthcare professionals and supported the clinical management. The DASH questionnaire was able to capture the patient’s experience with the injury and surgical recovery, corroborating an improvement in function while there was persistent post-surgical pain.

## Background

Patients bring their own experience, expectations and expertise to their care [1, 2]. The provision of personalized care is one of the main goals of healthcare systems [3], and patient-reported outcomes can facilitate that. Patient-reported outcomes include reports that come directly from the patients without the interpretation of their care providers or caregiver [4, 5].

Patient-reported outcome measure (PROMs) are concerned with the outcomes of a patient’s health condition or disability, including measures of symptom burden that report the frequency, severity, and impact of symptoms [6–12]. A diverse group of measures fall under the PROMs umbrella, including psychological/emotional health, adverse events and symptoms, and functioning [6–12]. PROMs can be used at the individual level in routine clinical care and at the health care system level.

Routine use of PROMs in clinical practice can provide benefits for patient management, including facilitating patient–clinician communication, specifically about issues that are important to patients, facilitating communication among health professionals, promoting shared decision making, and monitoring the progression of a patient’s illness and response to treatment plans [6–14]. PROMs can be used to track progress, monitor changes in health, and adjust treatment and the frequency of clinic visits [14–16].

The patient is the one who experiences both the processes and the outcomes of care. Information held by the patient is vital for clinical and self-management, improving health outcomes, delivery of care, organization of health systems, and formulation of health policies. Patient-reported outcome measures (PROMs) can play an important role in supporting a patient’s self-management. However, we know little about use of PROMs by patients to managing health.

This short report describes a patient-led use of a PROM in the management of a rotator cuff injury.

### Patient narrative (one of us, DJT)

#### The accident

At the end of a yoga class, I asked my instructor to review a warrior’s position. Not on a sticky mat, I slipped and reached out with my right arm. Both my instructor and I fell on my outstretched arm. Two weeks after this accident, I could not drive a car, I could not lift a coffee cup with my right arm, and I could not cut my food.

#### Self-management

I went to a physiotherapist and began rehabilitation exercises. These were primarily shoulder strengthening exercises. Physiotherapy improved my ability to function; I could lift my cup of coffee again. However, the pain was not subsiding, so I went to my primary care physician. The resident on rotation ordered an X-ray and an ultrasound examination and prescribed a synthetic opioid with acetaminophen. Three months after the accident, the ultrasound examination revealed a “full-thickness tear of the supraspinatus, 2.0 x 1.5 cm.”

#### Patient experience using the disabilities of the arm, shoulder and hand

**Fig. 1:**
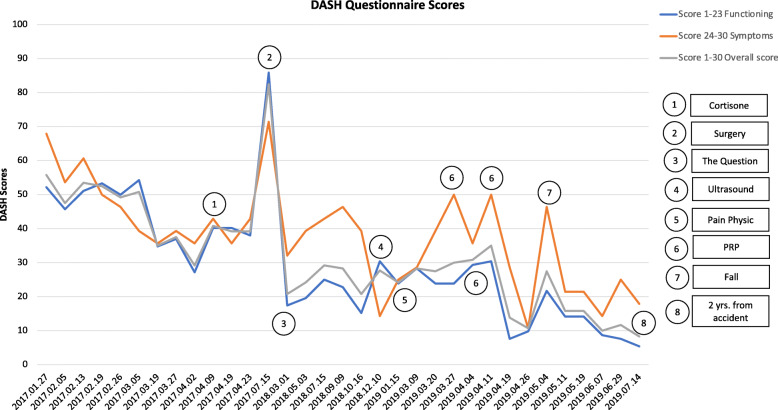
Disability of Arm, Shoulder and Hand (DASH) scores 3 months to 2 years after the accident

At that time, I found the Disabilities of the Arm, Shoulder and Hand (DASH) on-line [17, 18]. The DASH is a patient-reported outcome measure that includes 30 items with five response options for each and measures physical function and symptoms. The DASH was designed for patients with musculoskeletal disorders of the upper limb, patients like me. I chose it because the questions were personally relevant and easy to answer. Also the physiotherapist was familiar with this measure. I started using the DASH once a week to monitor my rehabilitation with my physiotherapist to support the management of physical functioning and pain. See Table 1 and Fig. 1 for the DASH scores from 3 months to 2 years 9 months following the accident.

**Table 1:** Disabilities of the Arm, Shoulder and Hand (DASH) Questionnaire and Scores. Data presented from three months after the accident to two years after

Dates	DASH 1–23	DASH 24–30	DASH 1–30	Score 1–23	Score 24–30	Score 1–30	Main Events through Recovery
2016.10.16							**The accident**
2017.01.27	**71**	**26**	**97**	**52.2**	**67.9**	**55.8**	**3-months after the accident**
2017.02.05	65	22	87	45.7	53.6	47.5	
2017.02.13	70	24	94	51.1	60.7	53.5	
2017.02.19	72	21	93	53.3	50.0	52.5	
2017.02.26	69	20	89	50.0	46.4	49.2	
2017.03.05	73	18	91	54.3	39.3	50.8	
2017.03.19	55	17	72	34.8	35.7	35	
2017.03.27	57	18	75	37.0	39.3	37.5	
2017.04.02	48	17	65	27.2	35.7	29.2	
2017.04.09	**60**	**19**	**79**	**40.2**	**42.9**	**40.8**	**1**^a^**Cortisone**
2017.04.19	60	17	77	40.2	35.7	39.2	
2017.04.23	58	19	77	38.0	42.9	39.2	
2017.07.15	**102**	**27**	**129**	**85.9**	**71.4**	**82.5**	**2**^a^**Surgery**
2018.03.01	**39**	**16**	**55**	**17.4**	**32.1**	**20.8**	**3**^a^**The Question**
2018.05.03	41	18	59	19.6	39.3	24.2	
2018.07.15	46	19	65	25.0	42.9	29.2	
2018.09.09	44	20	64	22.8	46.4	28.3	
2018.10.16	37	18	55	15.2	39.3	20.8	
2018.12.10	**51**	**11**	**62**	**30.4**	**14.3**	**27.7**	**4**^a^**Ultrasound**
2019 01.15	**45**	**14**	**59**	**23.9**	**25.0**	**24.2**	**5**^a^**Pain Physic**
2019.03.09	49	15	64	28.3	28.6	28.3	
2019.03.20	45	18	63	23.9	39.3	27.5	
2019.03.27	**45**	**21**	**66**	**23.9**	**50.0**	**30**	**6**^a^**PRP**^b^**-wk 1**
2019.04.04	**50**	**17**	**67**	**29.3**	**35.7**	**30.8**	**6**^a^**PRP-wk 2**
2019.04.11	**51**	**21**	**72**	**30.4**	**50.0**	**35**	**6**^a^**PRP-wk 3**
2019.04.19	30	15	45	7.6	28.6	13.8	Four weeks after PRP
2019.04.26	32	10	42	9.8	10.7	10.7	Five weeks after PRP
2019.05.04	**43**	**20**	**63**	**21.7**	**46.4**	**27.5**	**7**^a^**Fell**
2019.05.11	36	13	49	14.1	21.4	15.8	
2019.05.19	36	13	49	14.1	21.4	15.8	
2019.06.07	31	11	42	8.7	14.3	10	
2019.06.29	30	14	44	7.6	25.0	11.7	
2019.07.14	**28**	**12**	**40**	**5.4**	**17.9**	**8.3**	**8**^a^**Two years**

NOTE: ^a^These numbers correspond with the ones on Fig. 1; ^b^*PRP* Platelet-rich plasma injections

Two months later, magnetic resonance imaging showed a completely torn tendon with 3 cm of retraction. Over 3 months of conservative treatment my total DASH score went from a high level of disability of 56 to 39 (17 point change). However, the score of 39 was much higher than the general population normative score of 10 [18]. I stopped doing the DASH 2 weeks after having a cortisone injection about 3 months before the surgery, because I was in more pain and felt that completing the DASH wasn’t going to add much to my actual health state and management.

Nine months after the accident, my surgeon performed an arthroscopic repair of the supraspinatus without biceps tenodesis because the biceps anchor was intact. His post-operative orders were shoulder immobilization for 9 weeks with passive range of motion exercises for 6 weeks, assisted active range of motion at 8 weeks and strengthening at 10 weeks. At 15 weeks I was still having anterior shoulder pain that interfered with physiotherapy exercises and enjoyment of my normal activities. I was taking two or three painkillers a day, whereas before surgery I was taking one at night. My surgeon felt there was good range and strength with abduction, so the “rotator cuff repair has done its job in that regard.”

Six months post-surgery (15 months after the accident) when I saw my surgeon again because of pain, my range of motion was very close to normal, and I was regaining more function. However, I was still experiencing pain and disability in my hand and shoulder that limited my exercise intensity and activities such as hiking, doing garden and housework, and using the computer. My physiotherapist had initiated intramuscular stimulation of pectoral, bicep, upper trapezius and cervical spine to relieve the pain.

My surgeon asked me: “Are you feeling better than you were before surgery?” My mind circled around the question, trying to remember how I was before the surgery 6 months earlier. I had to answer: “I don’t know.” Later I remembered that I had been filling out the DASH questionnaire and could compare my disability over time to answer my surgeon’s question. I had the longitudinal data needed to answer the question. A month later I completed the DASH and was surprised to see how much the score had improved, going down from 39 before surgery to 21.

### Using the disabilities of the arm, shoulder and hand for self-management during recovery

I had learned that the DASH has been widely used [17–20]. From what I read it appeared that the DASH was reliable and measured what it was supposed to measure. Supporting documents on-line indicated a score of 10 (SD of 15) is the average DASH score for healthy people (larger score is worse) [17]. Also, I learned that 15-point difference in scores measuring before and after was considered a “clinically important change.” [17] This information helped me to interpret my scores as I went through recovery.

Although the DASH scoring wasn’t intended to separate Questions 1–23 from Question 24–30, I looked at my score for the questions related to physical function and my score for the questions asking about pain (symptoms) in addition to my overall score. I used Excel to keep track of my data (Table 1) and create the figure (Fig. 1). This allowed me to see that I was experiencing pain, even though my range of motion and function had improved.

Sharing these results with my primary care provider resulted in a cascade of effects, including ordering an ultrasound and referral to a pain management specialist and to the regional pain clinic. The ultrasound identified tendinopathy of the supraspinatus and the subscapularis (diffuse heterogeneity, not inflammation). The pain management specialist provided both mindfulness therapy (meditation and visualization) and two and a half months later platelet-rich plasma injections (PRP). Four weeks after the PRP treatment, both function and pain had improved.

In conclusion, I found that the ability to assess outcomes during pre- and post-surgical rehabilitation was facilitated by using the DASH. The DASH questionnaire was able to capture my experience with the injury and surgery recovery, corroborating that physiotherapy prior to surgery helped to stabilize function and pain. Surgery improved function over a six-month period, but I continued to have persistent pain. Communication with my healthcare providers led to ultrasound examination and referral to a pain specialist.

DASH scores improved from 56 3 months after the accident to 8 2 years after surgery. My feeling was that function (range of motion, strength) had improved, while pain had not changed over a year after surgery. DASH scores for Questions 1–23 (function) did show a steady improvement over time, but DASH scores for 24–30 (pain) benefited from non-surgical pain management. I continue to have periods of pain that interfere with my activities.

## Discussion

This paper describes a novel patient-led use of a PROM. The patient found and used a PROM to support self-management and enhance communication with healthcare providers. The patient took the initiative to look for a measure that could help monitor pre- and post-rehabilitation. The patient used the Google search engine to find the DASH [17]. The information included was sufficient for the patient to learn how to use the measure, score it and interpret the scores.

The ability to separate items on physical function and activities (1–23) and items on severity of symptoms/pain (24–30) was very helpful to the patient and her healthcare providers in managing her recovery. Later studies have reported on this multidimensionality of the DASH [21]. The extra information provided important and useful insights into the trajectories of recovery. Specifically, the serial concrete responses from the DASH confirmed the substantial improvement in physical function and importantly, highlighted the persistent burden of pain, giving the patient the confidence that the burden was real and that it should be brought to the attention of her healthcare providers.

The case described in this paper highlights how PROMs can be used to promote patient-centred care in which patients are empowered to self-manage. This case describes how the use can empower a patient in their own management to recovery after the accident and surgery. The use of PROMs in clinical practice has been a motivation originated from clinicians and researchers interested in using these measures to support care and improve outcomes [6–16]. Very little has been written about patient-led use of PROMs. This may be due to the fact that many developers of PROMs focused on the use of the measures by healthcare providers, researchers, healthcare managers and policy makers, rather than patient-led use of PROMs.

For instance, the developers of the DASH highlight that “*DASH gives clinicians and researchers the advantage of having a single, reliable instrument that can be used to assess any or all joints in the upper extremity*.” [18] However, in this case we described how a measure that was not intended to be used by a patient empowered the patient to use DASH longitudinal data, linking it to treatment changes and discussing physical functioning and symptoms burden throughout treatment, post-surgery, and recovery with her providers.

The use of DASH by the patient fits within the framework by Santana & Feeny [11] in which the completion of PROM and sharing the results with the providers can enhance communication, lead to referral to other specialists, additional testing, engage the patient in self-management, and ultimately improve patient-reported outcomes. Specifically, putting this case into the context of the framework, the patient completed the DASH and shared results with her primary care provider leading to a cascade of effects, including ordering an ultrasound that identified tendinopathies and referral to a pain management specialist and to the regional pain clinic. This new knowledge provided an explanation for the persistence of the pain and reduced anxiety about other potential causes of the persistent pain.

This case raises questions about patient-led PROM use. In a patient-centred model, patients are at the centre of the care, and care is tailored to their needs, values and preferences [1]. This patient-led case is an example in which the patient takes the initiative to find a PROM that could help her through her experience. Patients have different motivations, desires to be engaged in their self-management, and one-size doesn’t fit all. Patients ability to choose the right PROM, will depend on resources available, factors such as patient characteristics, level of education and social aspects. In this patient narrative, the patient is an emeritus professor with vast experience and expertise in health research. To promote the use of PROMs by patients, healthcare system could make PROMs available to patients as tools to support their management.

Perhaps a shift in policy is needed to make such instruments available to patients free of charge to support the patient-led PROM use. Potentially, clinics and systems could support the patient-led PROM use by developing strategies with patients, including co-designing training programs and information materials on using and interpreting these measures that were developed to capture what matters most to them. Future work is needed to enhance the individual use of PROMs. This patient narrative is a steppingstone towards the individual use.

In conclusion, the patient developed expertise in using the DASH. She found the DASH useful in monitoring her recovery before and after surgery. The DASH questionnaire was able to capture that physiotherapy prior to surgery helped to stabilize function and pain, and that while surgery improved function, there was a persistent level of pain that benefited from non-surgical management.

## Data Availability

◦The datasets used and/or analysed during the current study are available from the corresponding author on reasonable request.

## Abbreviations

DASH: The Disabilities of the Arm, Shoulder and Hand - the DASH questionnaire
PROMs: Patient-reported Outcome Measures
PRP: Platelet-rich Plasma injections
SD: Standard Deviation
