# Correction to: Patient-led use of patient-reported outcome measure in self-Management of a Rotator Cuff Injury

**DOI:** 10.1186/s41687-021-00340-y

**Published:** 2021-08-18

**Authors:** Maria J. Santana, Darrell J. Tomkins

**Affiliations:** 1 Departments of Pediatrics and Community Health Sciences, Cumming School of Medicine, University of Calgary, 3280 Hospital Drive NW, TRW Building, 3rd Floor, Calgary, Alberta T2N 4Z6 Canada; 2 Department of Medical Genetics, University of Alberta, Edmonton, Canada

**Correction to: Journal of Patient-Reported Outcomes 5, 8 (2021)**

**https://doi.org/10.1186/s41687-020-00283-w**

Following publication of the original article [1], the authors identified an error in Fig. 1.

The correct version of Fig. 1 is given below:

**Fig. 1:**
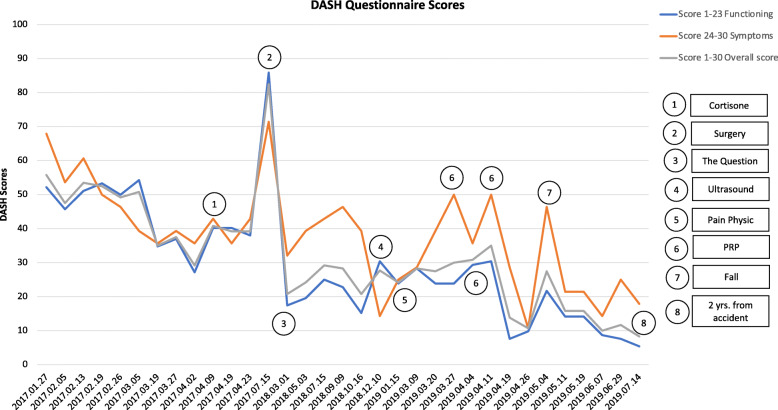
Disability of Arm, Shoulder and Hand (DASH) scores 3 months to 2 years after the accident

The original article [1] has been corrected.
